# Supplementary material for: The soil-borne white root rot pathogen Rosellinia necatrix expresses antimicrobial proteins during host colonization
Source: PLoS Pathog. 2024 Jan 18;20(1):e1011866. doi: 10.1371/journal.ppat.1011866 (PMC10796067; doi:10.1371/journal.ppat.1011866)
Supplement: S1 Table — (DOCX) [file ppat.1011866.s001.docx]

**S1 Table. Annotation of predicted effector proteins of *R. necatrix* strain R18.**

| **Query ID** | **Accession ID^a^** | **Annotation^b^** | **Organism^c^** | **Query coverage** | **E value** | **Identity (%)^d^** |
| --- | --- | --- | --- | --- | --- | --- |
| **Cell wall degrading enzymes** | | |  |  |  |  |
| FUN_005758 | XM_047973256.1 | 1,4-β-xylosidase | *Xylaria bambusicola* | 100% | 2E-140 | 77.49% |
| FUN_004100 | XM_047973055.1 | 1,4-β-xylosidase | *Xylaria bambusicola* | 99% | 1E-123 | 85.37% |
| FUN_007661 | XM_047977209.1 | 1,4-β-xylosidase | *Xylaria bambusicola* | 99% | 4E-129 | 85.85% |
| FUN_007704 | XM_047978660.1 | Acetylxylan esterase A | *Xylaria bambusicola* | 90% | 0 | 91.73% |
| FUN_003106 | XM_047976713.1 | Carbohydrate esterase family 4 | *Xylaria bambusicola* | 100% | 0 | 78.52% |
| FUN_001755 | XM_047979347.1 | Carbohydrate esterase family 4 | *Xylaria bambusicola* | 100% | 4E-162 | 89.27% |
| FUN_010853 | XM_047976371.1 | Cellulose monooxygenase | *Xylaria bambusicola* | 100% | 4E-131 | 87.32% |
| FUN_008445 | XM_047977156.1 | Cellulose monooxygenase | *Xylaria bambusicola* | 100% | 5E-153 | 87.39% |
| FUN_001148 | XM_046148909.1 | Cutinase | *Microdochium trichocladiopsis* | 100% | 8E-75 | 54.39% |
| FUN_001573 | XM_047972062.1 | Cutinase-like protein | *Xylaria bambusicola* | 91% | 2E-97 | 81.18% |
| FUN_005500 | XM_047972798.1 | Glycoside hydrolase family 10 | *Xylaria bambusicola* | 98% | 0 | 82.68% |
| FUN_000721 | XM_047979386.1 | Glycoside hydrolase family 12 | *Xylaria bambusicola* | 98% | 2E-120 | 73.27% |
| FUN_002439 | XM_047969089.1 | Glycoside hydrolase family 16 | *Xylaria bambusicola* | 100% | 1E-150 | 88.26% |
| FUN_002410 | XM_048004698.1 | Glycoside hydrolase family 16 | *Daldinia vernicosa* | 100% | 2E-110 | 58.33% |
| FUN_010054 | XM_047977697.1 | Glycoside hydrolase family 17 | *Xylaria bambusicola* | 98% | 0 | 85.76% |
| FUN_008369 | XM_047932653.1 | Glycoside hydrolase family 43 | *Daldinia caldariorum* | 100% | 0 | 79.27% |
| FUN_000284 | XM_047979212.1 | Glycoside hydrolase family 43 | *Xylaria bambusicola* | 100% | 0 | 79.88% |
| FUN_008666 | XM_047975058.1 | Glycoside hydrolase family 43 | *Xylaria bambusicola* | 100% | 0 | 82.72% |
| FUN_010499 | XM_047978711.1 | Glycoside hydrolase family 45 | *Xylaria bambusicola* | 99% | 2E-133 | 85.24% |
| FUN_007581 | XM_047971870.1 | Glycoside hydrolase family 5 | *Xylaria bambusicola* | 99% | 0 | 84.94% |
| FUN_004897 | XM_047976082.1 | Lytic polysaccharide monooxygenase | *Xylaria bambusicola* | 100% | 3E-126 | 84.11% |
| FUN_007011 | XM_022614464.1 | Mutanase | *Colletotrichum orchidophilum* | 98% | 3E-48 | 70.00% |
| FUN_008395 | XM_047972336.1 | O-acetylxylan esterase | *Xylaria bambusicola* | 100% | 3E-141 | 89.33% |
| FUN_007837 | XM_040856674.1 | Pectate lyase | *Pseudomassariella vexata* | 100% | 6E-129 | 84.11% |
| FUN_004715 | XM_047972211.1 | Polysaccharide lyase | *Xylaria bambusicola* | 100% | 4E-154 | 87.71% |
| FUN_001279 | XM_047975706.1 | Polysaccharide lyase family 7 protein | *Xylaria bambusicola* | 100% | 4E-133 | 82.38% |
| FUN_009124 | XM_047969579.1 | Putative endo-1,3(4)-beta-glucanase | *Xylaria bambusicola* | 99% | 2E-142 | 78.03% |
| FUN_010529 | XM_047974852.1 | Xylanase | *Xylaria bambusicola* | 100% | 7E-129 | 91.41% |
| FUN_009151 | XM_047976406.1 | Lysozyme-like domain-containing protein | *Xylaria bambusicola* | 99% | 1E-95 | 83.73% |
| FUN_003275 | XM_018318865.1 | Muramidase | *Purpureocillium lilacinum* | 92% | 2E-97 | 64.29% |
| FUN_005762 | XM_047972660.1 | Putative muramidase | *Xylaria bambusicola* | 99% | 1E-102 | 71.16% |
| FUN_001575 | XM_047978226.1 | Chloroperoxidase | *Xylaria bambusicola* | 99% | 2E-133 | 76.17% |
| **Carbohydrate binding** | |  |  |  |  |  |
| FUN_003047 | XM_047979309.1 | Concanavalin A-like lectin | *Xylaria bambusicola* | 99% | 1E-176 | 81.06% |
| FUN_003763 | XM_047969123.1 | WSC domain-containing protein | *Xylaria bambusicola* | 88% | 3E-121 | 77.39% |
| FUN_000265 | XM_047976926.1 | WSC domain-containing protein | *Xylaria bambusicola* | 100% | 0 | 79.34% |
| **Lysin motif (LysM) effectors** | |  |  |  |  |  |
| FUN_000206 | XM_001268710.1 | LysM domain-containing protein | *Aspergillus clavatus* | 83% | 4E-08 | 38.46% |
| FUN_006904 | XM_001268710.1 | LysM domain-containing protein | *Aspesgillus clavatus* | 98% | 8E-25 | 37.84% |
| **Hydrophobins** | |  | | | | |
| FUN_007007 | XM_040863415.1 | Cerato-ulmin family protein | *Pseudomassariella vexata* | 81% | 1E-32 | 76.71% |
| FUN_000192 | XM_047977015.1 | Cerato-ulmin family protein | *Xylaria bambusicola* | 95% | 5E-40 | 82.28% |
| FUN_001605 | XM_047978428.1 | Hydrophobin | *Xylaria bambusicola* | 100% | 1E-31 | 57.55% |
| FUN_011531 | XM_047976151.1 | Hydrophobin-like protein | *Xylaria bambusicola* | 84% | 6E-40 | 90.14% |
| **Proteases** |  |  |  |  |  |  |
| FUN_001370 | XM_047978948.1 | Acid protease | *Xylaria bambusicola* | 100% | 7E-106 | 65.82% |
| FUN_004271 | XM_036641540.1 | Neutral protease 2-like protein | *Colletotrichum siamense* | 99% | 1E-114 | 51.81% |
| FUN_000625 | XM_033580201.1 | Papain inhibitor | *Daldinia childiae* | 97% | 2E-36 | 65.12% |
| **Toxins** | | | |  |  |  |
| FUN_002843 | XM_047969234.1 | Necrosis inducing protein-domain-containing protein | *Xylaria bambusicola* | 100% | 6E-147 | 82.45% |
| FUN_005483 | XM_046238898.1 | Necrosis inducing protein-domain-containing protein | *Ilyonectria robusta* | 92% | 3E-82 | 54.20% |
| FUN_010366 | XM_047972065.1 | Cerato-platanin family protein | *Xylaria bambusicola* | 100% | 2E-64 | 80.83% |
| **Others** |  |  |  |  |  |  |
| FUN_000283 | XM_047980145.1 | Deoxyribonuclease NucA/NucB | *Xylaria bambusicola* | 99% | 1E-124 | 81.73% |
| FUN_003451 | XM_047976696.1 | Guanine-specific ribonuclease N1 | *Xylaria bambusicola* | 100% | 3E-61 | 79.82% |
| FUN_009264 | XM_047973582.1 | emp24/gp25L/p24 family/GOLD-domain-containing protein | *Xylaria bambusicola* | 100% | 9E-108 | 75.37% |
| FUN_009552 | XM_047973428.1 | emp24/gp25L/p24 family/GOLD-domain-containing protein | *Xylaria bambusicola* | 100% | 4E-127 | 88.61% |
| FUN_005699 | XM_047975004.1 | emp24/gp25L/p24 family/GOLD-domain-containing protein | *Xylaria bambusicola* | 100% | 4E-123 | 90.96% |
| FUN_001432 | XM_047970391.1 | 2OG-Fe(II) oxygenase superfamily protein | *Xylaria bambusicola* | 100% | 5E-133 | 75.95% |
| FUN_011359 | XM_048011353.1 | FMN-dependent alpha-hydroxy acid dehydrogenase | *Daldinia vernicosa* | 95% | 2E-69 | 74.10% |
| FUN_011399 | KY782142.1 | Heme-thiolate peroxidase | *Ustulina deusta* | 99% | 1E-124 | 73.33% |
| FUN_001799 | XM_047970313.1 | Intradiol ring-cleavage dioxygenase | *Xylaria bambusicola* | 100% | 0 | 74.93% |
| FUN_009516 | XM_049306200.1 | Putative choline dehydrogenase | *Daldinia loculata* | 87% | 3E-28 | 63.10% |
| FUN_004320 | XM_047974920.1 | ERV/ALR sulfhydryl oxidase | *Xylaria bambusicola* | 91% | 1E-87 | 79.49% |
| FUN_002834 | XM_047975475.1 | Acyl transferase | *Xylaria bambusicola* | 76% | 1E-93 | 78.57% |
| FUN_006056 | XM_047978164.1 | Apc13p ubiquitin ligase | *Xylaria bambusicola* | 87% | 4E-59 | 65.41% |
| FUN_005907 | XM_047975260.1 | Cyanovirin-N | *Xylaria bambusicola* | 93% | 1E-57 | 66.94% |
| FUN_006163 | XM_046160209.1 | Cyclophilin-like protein | *Microdochium trichocladiopsis* | 100% | 1E-102 | 81.67% |
| **Unknown** |  |  |  |  |  |  |
| FUN_010039 | XM_033580564.1 | Bys1 domain-containing protein | *Daldinia childiae* | 100% | 5E-79 | 82.09% |
| FUN_005325 | XM_047969535.1 | Met-10+ like-protein-domain-containing protein | *Xylaria bambusicola* | 100% | 1E-95 | 70.79% |

**^a^**Database accession ID where subject was deposited.

**^b^**Annotation of the best hit using BLAST (tblastn).

**^c^**Organism where the homolog was annotated.

**^d^**Percentage of identity of the query.
