# Supplementary material for: The soil-borne white root rot pathogen Rosellinia necatrix expresses antimicrobial proteins during host colonization
Source: PLoS Pathog. 2024 Jan 18;20(1):e1011866. doi: 10.1371/journal.ppat.1011866 (PMC10796067; doi:10.1371/journal.ppat.1011866)
Supplement: S2 Table — (DOCX) [file ppat.1011866.s002.docx]

**S2 Table.** **Single nucleotide polymorphism ratios (SNPs in %) and presence-absence variation for predicted effector genes in the *R. necatrix* strains sequenced in this study using *R. necatrix* strain R18 as a reference.**

| **Effector in *R. necatrix* strain R18 (gene ID)** | **Homologs in other *R. necatrix* strains^*^** | | | | | | | |
| --- | --- | --- | --- | --- | --- | --- | --- | --- |
|  | **Rn19** | **CH12** | **Rn400** | **R10** | **R25** | **R27** | **R28** | **R30** |
| FUN_000053 | 3.29 | 3.29 | 4.15 | 0.00 | 0.17 | 0.00 | 0.00 | 0.17 |
| FUN_000192 | 3.63 | 3.29 | 3.29 | 0.00 | 0.17 | 0.00 | 0.00 | 0.17 |
| FUN_000206 | 3.98 | 3.81 | 3.81 | 0.17 | 0.17 | 0.17 | 0.00 | 0.17 |
| FUN_000251 | 3.98 | 3.98 | 3.98 | 0.00 | 0.35 | 0.87 | 0.87 | 0.35 |
| FUN_000265 | 8.82 | 8.65 | 8.82 | 0.00 | 1.04 | 1.21 | 1.21 | 1.04 |
| FUN_000283 | 6.06 | 6.06 | 5.36 | A | 1.56 | 1.56 | 1.38 | 1.56 |
| FUN_000284 | 7.61 | 7.61 | 8.13 | 0.35 | 1.04 | 1.56 | 1.56 | 1.04 |
| FUN_000359 | 3.46 | 4.50 | 3.11 | 0.00 | 1.90 | 1.56 | 1.56 | 1.56 |
| FUN_000367 | 2.25 | 2.25 | 2.25 | 0.00 | 0.17 | 0.00 | 0.00 | 0.17 |
| FUN_000625 | 2.60 | 2.60 | 2.60 | 0.00 | 0.69 | 0.00 | 0.00 | 0.69 |
| FUN_000721 | 3.81 | 3.81 | 1.21 | 0.00 | 1.04 | 0.00 | 0.17 | 1.04 |
| FUN_000856 | 2.42 | 2.60 | 2.60 | 0.17 | 0.87 | 0.35 | 0.35 | 1.04 |
| FUN_000873 | 2.77 | 2.77 | 2.77 | 0.00 | 0.87 | 0.87 | 0.87 | 0.87 |
| FUN_000886 | 4.33 | 7.09 | 4.33 | 0.00 | 1.38 | 1.21 | 1.21 | 1.56 |
| FUN_000960 | 7.61 | 7.61 | 8.65 | 0.52 | 2.08 | 1.73 | 1.38 | 1.90 |
| FUN_001031 | 2.42 | 3.29 | 2.42 | 0.17 | 0.69 | 2.94 | 3.29 | 0.69 |
| FUN_001148 | 3.29 | 3.29 | 3.29 | 0.00 | 1.21 | 0.52 | 0.52 | 1.04 |
| FUN_001231 | 2.25 | 2.25 | 2.42 | 0.00 | 0.17 | 0.00 | 0.00 | 0.17 |
| FUN_001279 | 6.75 | 6.75 | 6.75 | 0.00 | 1.73 | 1.73 | 0.87 | 1.73 |
| FUN_001370 | 8.13 | 7.61 | 7.96 | 0.00 | 2.08 | 2.08 | 0.87 | 2.08 |
| FUN_001432 | 5.54 | 5.19 | 5.19 | 0.00 | 1.38 | 1.90 | 0.87 | 1.38 |
| FUN_001497 | 2.42 | 2.60 | 2.77 | 0.00 | 0.00 | 0.35 | 0.69 | 0.00 |
| FUN_001504 | 3.29 | 3.11 | 3.29 | 0.00 | 0.00 | 0.00 | 0.00 | 0.00 |
| FUN_001515 | 2.08 | 2.08 | 2.08 | 0.00 | 0.69 | 0.00 | 0.00 | 0.87 |
| FUN_001527 | 5.88 | 6.23 | 5.88 | 3.98 | 1.38 | 6.06 | 0.00 | 6.23 |
| FUN_001573 | 4.50 | 4.50 | 4.50 | 0.00 | A | 0.00 | 0.00 | 0.69 |
| FUN_001575 | 6.06 | 5.88 | 5.88 | 0.00 | 1.38 | 0.00 | 0.00 | 1.38 |
| FUN_001585 | 4.67 | 4.84 | 3.98 | 0.00 | 0.87 | 0.17 | 0.00 | 0.69 |
| FUN_001595 | 2.08 | 2.25 | 2.25 | 0.52 | 0.52 | 0.00 | 0.00 | 0.52 |
| FUN_001605 | 9.86 | 9.86 | 9.86 | 0.00 | 3.11 | 0.00 | 0.00 | 2.94 |
| FUN_001636 | 2.94 | 2.94 | 2.77 | 0.00 | 0.00 | 0.00 | 0.00 | 0.00 |
| FUN_001689 | 6.57 | 6.57 | 6.92 | 0.17 | 2.60 | 0.00 | 0.00 | 2.60 |
| FUN_001755 | 9.17 | 3.46 | 3.29 | 0.00 | 1.04 | 0.69 | 0.00 | 0.87 |
| FUN_001798 | 9.69 | 9.52 | 9.17 | 0.00 | 0.00 | 0.00 | 0.00 | 0.00 |
| FUN_001799 | 1.56 | 0.52 | 0.87 | 0.17 | 0.17 | 0.00 | 0.00 | 0.00 |
| FUN_001917 | 2.42 | 2.42 | 1.90 | 0.00 | 0.52 | 0.00 | 0.00 | 0.52 |
| FUN_002005 | 4.84 | 4.67 | 4.15 | 0.17 | 0.35 | 2.08 | 2.08 | 0.35 |
| FUN_002053 | 1.04 | 1.04 | 1.04 | 0.00 | 0.17 | 0.35 | 0.17 | 0.35 |
| FUN_002114 | 3.46 | 3.29 | 3.29 | 0.00 | 0.87 | 0.35 | 0.35 | 0.87 |
| FUN_002120 | 4.50 | 4.50 | 3.46 | 0.00 | 0.87 | 1.21 | 1.21 | 0.87 |
| FUN_002121 | 4.50 | 4.50 | 3.46 | 0.00 | 0.00 | 0.00 | 1.21 | 0.00 |
| FUN_002234 | 3.11 | 2.60 | 2.77 | 0.00 | 0.35 | 0.52 | 0.52 | 0.35 |
| FUN_002337 | 8.13 | 8.13 | 8.13 | 8.30 | 0.52 | 0.00 | 0.00 | 0.52 |
| FUN_002389 | 0.69 | 0.69 | 0.87 | 0.35 | 0.52 | 0.69 | 0.52 | 0.52 |
| FUN_002410 | 5.88 | 5.88 | 5.88 | 0.00 | 2.08 | 1.90 | 1.90 | 2.08 |
| FUN_002439 | 11.76 | 11.76 | 11.76 | 0.00 | 2.77 | 2.60 | 0.00 | 2.77 |
| FUN_002458 | 2.42 | 2.42 | 2.42 | 0.17 | 0.35 | 1.56 | 0.35 | 0.35 |
| FUN_002489 | 4.33 | 5.02 | 5.02 | 0.17 | 1.56 | 0.87 | 1.04 | 1.56 |
| FUN_002758 | 1.21 | 1.38 | 1.38 | 0.00 | 0.17 | 1.04 | 1.04 | 0.17 |
| FUN_002834 | 3.11 | 2.94 | 3.29 | 0.00 | 0.69 | 0.69 | 0.69 | 0.69 |
| FUN_002843 | 5.71 | 5.71 | 5.71 | 0.00 | 0.52 | 1.04 | 1.04 | 0.52 |
| FUN_003003 | 1.38 | 1.38 | 1.38 | 0.00 | 0.17 | 0.00 | 0.00 | 0.17 |
| FUN_003018 | 1.38 | 0.69 | 0.69 | 0.00 | 0.00 | 0.00 | 0.00 | 0.00 |
| FUN_003047 | 4.50 | A | 4.33 | 0.00 | 0.00 | 0.00 | 0.00 | 0.00 |
| FUN_003077 | 2.77 | 2.77 | 3.29 | 0.00 | 0.17 | 0.35 | 0.35 | 0.17 |
| FUN_003106 | 9.52 | 9.69 | 9.52 | 0.00 | 0.00 | 0.87 | 0.87 | 0.00 |
| FUN_003133 | 2.77 | 2.25 | 1.90 | 0.52 | 1.90 | 0.00 | 0.52 | 1.90 |
| FUN_003175 | 3.81 | 3.63 | 3.81 | 0.35 | 0.17 | 0.17 | 0.17 | 0.17 |
| FUN_003209 | 3.63 | 3.46 | 3.29 | 0.00 | 0.00 | 0.00 | 0.00 | 1.21 |
| FUN_003212 | 4.67 | 4.67 | 4.67 | 0.00 | 0.35 | 0.35 | 0.35 | 3.46 |
| FUN_003275 | 2.94 | 2.77 | 2.77 | 0.00 | 0.00 | 0.00 | 0.00 | 0.00 |
| FUN_003286 | 3.46 | 3.46 | 3.46 | 0.00 | 0.00 | 0.00 | 0.00 | 0.00 |
| FUN_003451 | 3.63 | 3.63 | 3.63 | 0.00 | 0.35 | 0.52 | 0.52 | 0.35 |
| FUN_003513 | 1.38 | 1.38 | 1.38 | 0.00 | 0.00 | 0.35 | 0.35 | 0.00 |
| FUN_003763 | 4.67 | 4.67 | 4.67 | 0.35 | 0.17 | 0.17 | 0.00 | 0.17 |
| FUN_003889 | 8.13 | 8.30 | 8.13 | 0.00 | 2.94 | 0.00 | 0.00 | 2.94 |
| FUN_003947 | 9.17 | 8.82 | 8.82 | 0.00 | 0.69 | 0.00 | 0.00 | 0.87 |
| FUN_004082 | 4.15 | 4.15 | 4.15 | 0.17 | 0.00 | 0.00 | 0.00 | 0.00 |
| FUN_004100 | 5.54 | 5.54 | 5.88 | 0.00 | 0.35 | 0.52 | 0.52 | 0.35 |
| FUN_004157 | 4.67 | 1.21 | 6.40 | 0.52 | 7.96 | 7.96 | 0.00 | 4.84 |
| FUN_004244 | 5.36 | 7.44 | 4.50 | 0.00 | 1.38 | 0.00 | 0.00 | 1.38 |
| FUN_004254 | 1.73 | 1.90 | 1.73 | 0.35 | 0.00 | 0.35 | 0.00 | 0.17 |
| FUN_004271 | 3.63 | 3.63 | 3.46 | 0.00 | 0.17 | 0.00 | 0.00 | 0.17 |
| FUN_004305 | 5.71 | 5.88 | 5.54 | 0.17 | 0.00 | 0.87 | 0.69 | 0.00 |
| FUN_004320 | 3.46 | 3.46 | 3.46 | 0.00 | 1.04 | 1.38 | 1.38 | 1.04 |
| FUN_004483 | 4.15 | 4.15 | 4.15 | 0.00 | 0.00 | 1.04 | 1.04 | 0.00 |
| FUN_004569 | 2.42 | 2.77 | 2.77 | 0.00 | 2.60 | 2.08 | 1.90 | 2.60 |
| FUN_004715 | 1.90 | 1.73 | 1.90 | 0.00 | 0.35 | 0.35 | 0.35 | 0.35 |
| FUN_004770 | 4.15 | 4.15 | 4.15 | 0.00 | 0.00 | 0.00 | 0.00 | 0.00 |
| FUN_004897 | 4.67 | 4.33 | 4.50 | 0.17 | 0.00 | 0.00 | 0.00 | 0.00 |
| FUN_004961 | 4.15 | 4.15 | 4.33 | 0.00 | 0.35 | 0.17 | 0.00 | 0.35 |
| FUN_005189 | 5.36 | 1.38 | 5.19 | 0.00 | 0.00 | 0.69 | 0.87 | 0.00 |
| FUN_005222 | 4.84 | 4.67 | 4.67 | 0.00 | 0.00 | 0.00 | 0.00 | 0.00 |
| FUN_005300 | 4.67 | 1.90 | 5.36 | 0.00 | 1.21 | 1.21 | 1.21 | 1.21 |
| FUN_005325 | 0.87 | 1.38 | 1.38 | 0.00 | 0.35 | 1.04 | 1.04 | 0.35 |
| FUN_005445 | 1.56 | 1.90 | 1.73 | 0.00 | 0.17 | 0.00 | 0.00 | 0.17 |
| FUN_005483 | 2.77 | 4.84 | 5.02 | 0.69 | 2.94 | 2.94 | 2.08 | 2.60 |
| FUN_005500 | 4.67 | 3.46 | 6.06 | 0.69 | 3.46 | 1.90 | 2.08 | 3.81 |
| FUN_005538 | 4.33 | 3.63 | 3.63 | 0.17 | 1.04 | 1.21 | 1.21 | 1.04 |
| FUN_005543 | 5.54 | 0.00 | 5.02 | 0.00 | 1.21 | 0.00 | 0.00 | 0.00 |
| FUN_005544 | 7.27 | 7.27 | 7.96 | 0.00 | 0.35 | 0.87 | 0.87 | 0.35 |
| FUN_005699 | 3.46 | 2.94 | 2.94 | 0.00 | 0.17 | 0.00 | 0.00 | 0.17 |
| FUN_005740 | 1.21 | 1.21 | 1.21 | 0.00 | 0.35 | 0.00 | 0.00 | 0.35 |
| FUN_005758 | 6.75 | 4.33 | 4.33 | 0.17 | 1.90 | 4.15 | 0.17 | 4.15 |
| FUN_005762 | 3.29 | 3.46 | 3.29 | 0.00 | 0.17 | 0.00 | 0.17 | 0.17 |
| FUN_005785 | 4.50 | 4.50 | 4.33 | 0.00 | 0.35 | 0.00 | 0.00 | 0.35 |
| FUN_005787 | 3.63 | 3.63 | 3.63 | 0.00 | 0.35 | 0.00 | 0.00 | 0.35 |
| FUN_005834 | 3.11 | 3.11 | 3.11 | 0.00 | 0.69 | 0.00 | 0.00 | 0.69 |
| FUN_005847 | 8.30 | 8.30 | 8.13 | 0.00 | 0.00 | 0.00 | 0.00 | 0.00 |
| FUN_005850 | 4.15 | 4.84 | 3.46 | 0.00 | 0.52 | 0.00 | 0.00 | 0.52 |
| FUN_005883 | 4.33 | 4.50 | 4.67 | 0.00 | 0.87 | 0.87 | 0.87 | 0.87 |
| FUN_005907 | 4.33 | 4.15 | 4.33 | 0.00 | 0.52 | 0.00 | 0.00 | 0.52 |
| FUN_005940 | 5.19 | 5.19 | 4.67 | 0.00 | 0.00 | 2.60 | 2.60 | 0.00 |
| FUN_005952 | 4.84 | 3.98 | 4.33 | 0.00 | 0.00 | 0.69 | 0.69 | 0.00 |
| FUN_005989 | 5.88 | 6.57 | 6.06 | 0.00 | 1.04 | 0.17 | 0.17 | 0.87 |
| FUN_005998 | 6.06 | 6.23 | 5.88 | 0.00 | 0.35 | 0.17 | 0.00 | 0.35 |
| FUN_006007 | 8.13 | 8.30 | 7.61 | 0.00 | 0.00 | 2.77 | 2.77 | 0.00 |
| FUN_006010 | 4.15 | 4.33 | 3.81 | 0.00 | 0.17 | 0.35 | 0.35 | 0.17 |
| FUN_006056 | 3.63 | 3.63 | 3.81 | 0.00 | 0.00 | 0.17 | 0.00 | 0.00 |
| FUN_006163 | 3.98 | 1.90 | 4.15 | 0.00 | 0.17 | 1.90 | 1.90 | 0.17 |
| FUN_006164 | 3.98 | 1.90 | A | 0.00 | 0.00 | 1.73 | 0.00 | 0.17 |
| FUN_006190 | 5.36 | 5.88 | 5.71 | 0.00 | 6.06 | 0.69 | 6.23 | 0.00 |
| FUN_006412 | 6.40 | 4.67 | 4.67 | 0.00 | 1.04 | 0.52 | 1.04 | 1.04 |
| FUN_006481 | 3.29 | 2.08 | 3.29 | 0.00 | 0.17 | 0.52 | 0.52 | 0.17 |
| FUN_006483 | 3.98 | 4.33 | 4.67 | 0.17 | 0.17 | 0.52 | 0.35 | 0.17 |
| FUN_006775 | 8.65 | 8.65 | 8.65 | 0.00 | 0.87 | 0.35 | 0.35 | 0.87 |
| FUN_006835 | 2.42 | 2.42 | 2.77 | 0.00 | 1.04 | 1.56 | 1.38 | 1.04 |
| FUN_006841 | 5.36 | 4.84 | 5.36 | 0.00 | 0.52 | 0.87 | 0.87 | 0.52 |
| FUN_006904 | 6.23 | 6.23 | 7.09 | 0.00 | 0.69 | 0.00 | 0.00 | 0.69 |
| FUN_006905 | 4.67 | 4.50 | 6.06 | 0.00 | 0.00 | 0.17 | 0.00 | 0.00 |
| FUN_007007 | 5.88 | 5.88 | 7.27 | 0.00 | 1.38 | 0.00 | 0.00 | 1.38 |
| FUN_007011 | 2.42 | 2.60 | 1.73 | 0.00 | 0.17 | 0.00 | 0.00 | 0.17 |
| FUN_007012 | 6.06 | 5.71 | 6.23 | 0.00 | 0.35 | 0.00 | 0.00 | 0.35 |
| FUN_007129 | 4.84 | 5.36 | 5.36 | 0.00 | 0.52 | 0.00 | 0.00 | 0.52 |
| FUN_007254 | 4.33 | 4.33 | 4.50 | 0.00 | 0.87 | 1.04 | 1.04 | 0.87 |
| FUN_007288 | 8.13 | 7.27 | 8.13 | 0.00 | 1.38 | 0.87 | 0.87 | 1.38 |
| FUN_007567 | 2.77 | 2.94 | 2.60 | 0.00 | 0.00 | 0.17 | 0.17 | 0.00 |
| FUN_007581 | 7.44 | 7.27 | 6.92 | 0.00 | 0.35 | 0.52 | 0.52 | 0.35 |
| FUN_007661 | 4.67 | 3.98 | 4.15 | 0.00 | 0.69 | 0.87 | 0.87 | 0.69 |
| FUN_007684 | 6.75 | 7.27 | 6.57 | 0.00 | 0.00 | 1.38 | 1.38 | 0.17 |
| FUN_007704 | 6.23 | 6.40 | 5.71 | 0.00 | 0.00 | 0.52 | 0.69 | 0.00 |
| FUN_007825 | 2.42 | 2.42 | 2.42 | 0.00 | 0.00 | 1.56 | 1.56 | 0.00 |
| FUN_007837 | 4.84 | 1.73 | 5.02 | 0.17 | 0.69 | 1.04 | 1.21 | 0.69 |
| FUN_007838 | 1.21 | 1.04 | 1.21 | 0.00 | 0.00 | 0.35 | 0.17 | 0.00 |
| FUN_007850 | 2.08 | 1.21 | 2.08 | 0.00 | 0.00 | 0.00 | 0.00 | 0.35 |
| FUN_007851 | 3.63 | 2.08 | 3.63 | 0.00 | 0.52 | 0.17 | 0.17 | 0.52 |
| FUN_007855 | 5.19 | 3.63 | 5.71 | 0.00 | 0.69 | 0.52 | 0.52 | 0.69 |
| FUN_007860 | 6.40 | 5.19 | 6.40 | 0.00 | 2.25 | 1.73 | 1.56 | 2.25 |
| FUN_007991 | 1.56 | 6.40 | 1.73 | 0.17 | 0.69 | 0.35 | 0.35 | 0.69 |
| FUN_008329 | 2.60 | 2.60 | 2.60 | 0.00 | 1.38 | 0.00 | 0.00 | 1.38 |
| FUN_008352 | 2.42 | 2.42 | 2.42 | 0.00 | 1.04 | 0.00 | 0.00 | 1.04 |
| FUN_008369 | 6.40 | 6.23 | 6.40 | 0.00 | 0.69 | 0.00 | 0.00 | 0.69 |
| FUN_008395 | 3.63 | 2.94 | 3.63 | 0.00 | 1.21 | 0.00 | 0.00 | 1.21 |
| FUN_008445 | 2.25 | 2.60 | 2.60 | 0.00 | 0.35 | 0.00 | 0.00 | 0.35 |
| FUN_008500 | 2.60 | 1.90 | 2.08 | 0.00 | 0.17 | 0.00 | 0.00 | 0.17 |
| FUN_008562 | 2.77 | 3.11 | 2.25 | 0.17 | 0.00 | 0.17 | 0.00 | 0.00 |
| FUN_008574 | 5.02 | 5.71 | 4.84 | 0.17 | 2.60 | 0.00 | 0.00 | 2.42 |
| FUN_008666 | 5.02 | 5.71 | 5.54 | 0.00 | 0.35 | 0.00 | 0.17 | 0.35 |
| FUN_008681 | A | A | A | 0.00 | 0.52 | 0.52 | 0.69 | 0.52 |
| FUN_008714 | 6.57 | 3.98 | 3.98 | 0.00 | 1.04 | 1.04 | 1.04 | 1.04 |
| FUN_008830 | 5.02 | 5.19 | 5.19 | 0.00 | 0.52 | 0.52 | 0.52 | 0.52 |
| FUN_009063 | 6.57 | 6.57 | 6.57 | 0.00 | 2.42 | 0.00 | 0.17 | 2.42 |
| FUN_009073 | 5.88 | 5.19 | 5.19 | 0.17 | 0.87 | 0.00 | 0.17 | 0.87 |
| FUN_009124 | 4.33 | 4.33 | 4.33 | 0.00 | 1.21 | 0.00 | 0.00 | 1.21 |
| FUN_009151 | 5.54 | 5.54 | 5.54 | 0.00 | 0.52 | 0.00 | 0.00 | 0.52 |
| FUN_009222 | 6.57 | 5.02 | 6.57 | 0.00 | 0.52 | 0.35 | 0.35 | 0.52 |
| FUN_009264 | 3.63 | 2.25 | 3.81 | 0.00 | 1.56 | 1.56 | 1.73 | 1.73 |
| FUN_009294 | 2.25 | 2.25 | 2.42 | 0.00 | 0.00 | 1.04 | 1.04 | 0.00 |
| FUN_009350 | 4.67 | 4.67 | 4.84 | 0.00 | 0.52 | 0.00 | 0.00 | 0.52 |
| FUN_009516 | 1.38 | 1.38 | 1.38 | 0.00 | 0.00 | 0.00 | 0.00 | 0.00 |
| FUN_009552 | 5.36 | 6.40 | 5.54 | 0.00 | 1.04 | 1.38 | 1.38 | 1.04 |
| FUN_009576 | 4.67 | 4.33 | 4.84 | 0.00 | 2.77 | 2.60 | 2.60 | 2.94 |
| FUN_009991 | 5.54 | 5.71 | 5.54 | 0.00 | 2.42 | 0.69 | 1.04 | 2.42 |
| FUN_010039 | 3.29 | 3.29 | 3.11 | 0.00 | 1.21 | 1.38 | 1.21 | 1.21 |
| FUN_010054 | 6.92 | 6.92 | 6.57 | 0.00 | 1.38 | 2.08 | 2.25 | 1.38 |
| FUN_010164 | A | A | A | 0.00 | 1.21 | 0.00 | 0.17 | 1.21 |
| FUN_010225 | 9.52 | 9.52 | 9.34 | 0.00 | 0.35 | 0.00 | 0.00 | 0.52 |
| FUN_010331 | 2.08 | 1.73 | 1.90 | 0.00 | 0.52 | 0.52 | 0.52 | 0.52 |
| FUN_010336 | 13.49 | 12.63 | 13.32 | 0.00 | 2.25 | 3.46 | 3.98 | 2.25 |
| FUN_010366 | 4.50 | 4.67 | 4.15 | 0.00 | 0.17 | 0.00 | 0.17 | 0.17 |
| FUN_010414 | 1.90 | 1.73 | 1.90 | 0.00 | 0.35 | 0.52 | 0.52 | 0.35 |
| FUN_010496 | 3.63 | 3.63 | 3.63 | 0.00 | 0.35 | 0.35 | 0.52 | 0.35 |
| FUN_010499 | 1.04 | 1.04 | 0.87 | 0.00 | 0.00 | 0.00 | 0.00 | 0.00 |
| FUN_010529 | 4.15 | 7.09 | 5.36 | 2.42 | 4.15 | 3.46 | 4.15 | 3.11 |
| FUN_010575 | 1.38 | 1.38 | 1.38 | 0.00 | 0.00 | 0.00 | 0.00 | 0.00 |
| FUN_010594 | 3.98 | 3.98 | 3.81 | 0.00 | 0.35 | 0.00 | 0.35 | 0.35 |
| FUN_010651 | 3.46 | 4.15 | 3.46 | 0.17 | 0.69 | 0.00 | 0.00 | 0.87 |
| FUN_010656 | 4.15 | 4.15 | 4.15 | A | 0.69 | 0.00 | 0.00 | 0.69 |
| FUN_010853 | 19.20 | 6.23 | 20.07 | 0.00 | 3.46 | 0.00 | 0.35 | 3.29 |
| FUN_010873 | 7.61 | 6.92 | 7.44 | 0.00 | 2.08 | 0.00 | 0.00 | 2.08 |
| FUN_010899 | 8.82 | 8.48 | 8.13 | 0.00 | 0.52 | 0.17 | 0.00 | 0.52 |
| FUN_010928 | 5.54 | 5.54 | 5.54 | 0.00 | 1.04 | 1.56 | 0.17 | 0.87 |
| FUN_011077 | 5.36 | 5.19 | 5.71 | 0.00 | 0.00 | 0.35 | 0.69 | 0.00 |
| FUN_011081 | 1.56 | 1.90 | 1.90 | 0.00 | 0.52 | 0.17 | 0.17 | 0.52 |
| FUN_011148 | 1.90 | 1.90 | 1.73 | 0.00 | 0.17 | 0.52 | 0.52 | 0.17 |
| FUN_011352 | 3.81 | 3.63 | 4.33 | 0.00 | 0.17 | 0.35 | 0.52 | 0.17 |
| FUN_011359 | 3.46 | 0.17 | 3.29 | 0.17 | 0.17 | 0.35 | 0.35 | 0.35 |
| FUN_011399 | 2.94 | 2.77 | 2.77 | 0.35 | 0.35 | 0.00 | 0.00 | 0.00 |
| FUN_011519 | 4.33 | 3.81 | 2.94 | 0.35 | 0.69 | 0.35 | 0.35 | 0.35 |
| FUN_011522 | 26.12 | 25.95 | 25.95 | 0.00 | 3.81 | 0.87 | 0.35 | 0.52 |
| FUN_011531 | A | A | A | 12.98 | A | 1.90 | 0.17 | 3.81 |
| FUN_011546 | 4.84 | 4.84 | 4.67 | 0.00 | 0.87 | 1.21 | 1.21 | 0.87 |

^*^ While grey boxes labelled with “A” indicate effector gene absence, numbers indicate the SNP ratio (%) in the homolog when compared with the effector gene in R. necatrix strain R18. Green cells indicate identical effector genes that lack SNPs when compared with the sequence in strain R18.
