# Supplementary material for: The soil-borne white root rot pathogen Rosellinia necatrix expresses antimicrobial proteins during host colonization
Source: PLoS Pathog. 2024 Jan 18;20(1):e1011866. doi: 10.1371/journal.ppat.1011866 (PMC10796067; doi:10.1371/journal.ppat.1011866)
Supplement: S3 Table — (DOCX) [file ppat.1011866.s003.docx]

**S3 Table. Annotated secondary metabolite clusters of *R. necatrix* strain R18.**

| **Contig** | **From** | **To** | **Most similar known cluster** | **Type** | **Organism** | **Similarity** | **Known activity^a^** |
| --- | --- | --- | --- | --- | --- | --- | --- |
| 4 | 28,584 | 63,895 | Swainsonine | Polyketide | *Alternaria oxytropis* | 33% | Phytotoxic [1] |
| 5 | 1,431,139 | 1,452,194 | Copalyl diphosphate | Terpene | *Diaporthe amygdali* | 42% | Super-elongation disease in plants [2,3] |
| 5 | 2,494,569 | 2,532,303 | Cytochalasin E/K | NRP+Polyketide | *Aspergillus clavatus* | 23% | Phytotoxic [4,5] |
| 6 | 409,707 | 450,119 | Wortmanamide A/B | NRP + Polyketide | *Talaromyces wortmannii* | 83% | Unknown [6] |
| 6 | 1,123,590 | 1,166,755 | Pyriculol | Polyketide | *Neurospora crassa* | 26% | Phytotoxic [7] |
| 6 | 3,495,713 | 3,545,733 | Apicidin | NRP | *Fusarium incarnatum* | 54% | Antiprotozoal activity [8] |
| 6 | 3,953,658 | 3,994,676 | Yanuthone D | Polyketide | *Aspergillus niger* | 20% | Antibiotic [9] |
| 6 | 4,187,217 | 4,235,557 | Iso-A82775C | Other | *Pestalotiopsis fici* | 41% | Antibacterial activity [10] |
| 7 | 2,248,951 | 2,299,047 | Enniatin | NRP | *Fusarium equiseti* | 100% | Phytotoxic [11] |
| 7 | 3,449,415 | 3,495,060 | Naphthalene | Polyketide | *Daldinia eschscholzii* | 33% | Phytotoxic [12] |
| 8 | 3,062,127 | 3,105,143 | Melanin | Polyketide | *Glarea lozoyensis* | 100% | Not involved in *R. necatrix* virulence [13] |
| 9 | 1,168,004 | 1,220,639 | Cytochalasin E/K | NRP+Polyketide | *Aspergillus clavatus* | 30% | Phytotoxic [4,5] |
| 10 | 2,744,211 | 2,797,043 | Fusaridione A | NRP + Polyketide | *Fusarium heterosporum* | 12% | Unknown [14] |

^a^References:

[1] Cook D, Donzelli BGG, Creamer R, Baucom DL, Gardner DR, Pan J, et al. Swainsonine biosynthesis genes in diverse symbiotic and pathogenic fungi. Genes, Genomes, Genetics. 2017;7: 1791-1797.

[2] Kawaide H. Biochemical and molecular analyses of gibberellin biosynthesis in fungi. Bioscience, Biotechnology, and Biochemistry. 2006;70: 583-590.

[3] Rademacher W, Graebe JE. Gibberellin A4 produced by *Sphaceloma manihoticola*, the cause of the super elongation disease of cassava (*Manihot esculenta*). Biochemical and Biophysical Research Communications. 1979;91: 35-40.

[4] Sawai K, Okuno T, Fujioka H, Furuya M. The relation between the phytotoxicity of Cythochalasin E and its molecular structure. Annals of the Phytopathological Society of Japan. 1983;49: 262-265.

[5] Thomas DD. Cytochalasin effects in plants and eukaryotic microbial systems. Frontiers in Biology. 1978;46: 257-275.

[6] Hai Y, Tang Y. Biosynthesis of long-chain N-acyl amide by a truncated PKS-NRPS hybrid megasynthase in fungi. Journal of the American Chemical Society. 2018;140: 1271-1274.

[7] Zhao Z, Ying Y, Hung Y, Tang Y. Genome mining reveals *Neurospora crassa* can produce the salicylaldehyde sordarial. Journal of Natural Products. 2019;82: 1029-1033.

[8] Darkin-Rattray SJ, Gurnett AM, Myers RW, Dulski PM, Crumley TM, Allocco JJ, et al. Apicidin: a novel antiprotozoal agent that inhibits parasite histone deacetylase. Proceedings of the National Academy of Sciences of the USA. 1996;93: 13143–13147.

[9] Holm DK, Petersen LM, Klitgaard A, Knudsen PB, Jarczynska ZD, Nielsen KF, et al. Molecular and chemical characterization of the biosynthesis of the 6-MSA-derived meroterpenoid yanuthone D in *Aspergillus niger*. Chemistry and Biology. 2014;21: 519-529.

[10} Pan Y, Liu L, Guan F, Li E, Jin J, Li J. et al. Characterization of a Prenyltransferase for Iso-A82775C biosynthesis and generation of new congeners of chloropestolides. ACS Chemical Biology. 2018;13: 703-711.

[11] Walton JD. Peptide phytotoxins from plant pathogenic fungi. In: Kleinkauf H, von Dohren H, editors. Biochemistry of peptide antibiotics. Berlin; 1990. pp. 179-203.

[12] Xu D, Xue M, Shen Z, Jia X, Hou X, Lai D, et al. Phytotoxic secondary metabolites from fungi. Toxins. 2021;13: 1-65.

[13] Shimizu T, Tsutae I, Kanematsu S. Functional analysis of a melanin biosynthetic gene using RNAi-mediated gene silencing in *Rosellinia necatrix*. Fungal Biology. 2014;118: 413-421.

[14] Kakule TB, Sardar D, Lin Z, Schmidt EW. Two related pyrrolidinedione synthetase loci in *Fusarium heterosporum* ATCC 74349 produce divergent metabolites. ACS Chemical Biology. 2013;8: 1549-1557.
