# Supplementary material for: The soil-borne white root rot pathogen Rosellinia necatrix expresses antimicrobial proteins during host colonization
Source: PLoS Pathog. 2024 Jan 18;20(1):e1011866. doi: 10.1371/journal.ppat.1011866 (PMC10796067; doi:10.1371/journal.ppat.1011866)
Supplement: S4 Table — (DOCX) [file ppat.1011866.s004.docx]

**S4 Table.** **Bacterial strains used in this study.**

| **Strain** | **Species** | **Family** | **Order** | **Medium** | **Gram** |
| --- | --- | --- | --- | --- | --- |
| R102 | *Pseudomonas knackmussii* | Pseudomonadaceae | Pseudomonadales | R2A | Negative |
| R19 | *Pseudomonas corrugata* | Pseudomonadaceae | Pseudomonadales | LBA | Negative |
| R103 | *Bacillus drentensis* | Bacillaceae | Bacillales | R2A | Positive |
| S7 | *Bacillus licheniformis* | Bacillaceae | Bacillales | TSA | Positive |
| R104 | *Paenarthrobacter ureafaciens* | Micrococcaceae | Micrococcales | R2A | Positive |
| S39 | *Arthrobacter enclensis* | Micrococcaceae | Micrococcales | R2A | Positive |
| R109 | *Ochrobactrum intermedium* | Rhizobiaceae | Rhizobiales | R2A | Negative |
| S26 | *Brucella ovis* | Rhizobiaceae | Rhizobiales | TSA | Negative |
| R30 | *Serratia ureilytica* | Enterobacteriaceae | Enterobacterales | TSA | Negative |
| S27 | *Enterobacter soli* | Enterobacteriaceae | Enterobacterales | TSA | Negative |
| R139 | *Microbacterium foliorum* | Microbacteriaceae | Micrococcales | TSA | Positive |
| S23 | *Microbacterium esteraromaticum* | Microbacteriaceae | Micrococcales | TSA | Positive |
| R121 | *Chryseobacterium indoltheticum* | Weeksellaceae | Flavobacteriales | TSA | Negative |
| Ri8 | *Chryseobacterium wanjuense* | Weeksellaceae | Flavobacteriales | TSA | Negative |
| R143 | *Cellulomonas soli* | Cellulomonadaceae | Micrococcales | R2A | Positive |
| R151 | *Paenibacillus lautus* | Paenibacillaceae | Paenibacillales | TSA | Positive |
| S6 | *Paenibacillus illinoisensis* | Paenibacillaceae | Paenibacillales | TSA | Positive |
| R155 | *Achromobacter denitrificans* | Alcaligenaceae | Burkholderiales | LBA | Negative |
| S72 | *Candidimonas bauzanensis* | Alcaligenaceae | Burkholderiales | TSA | Negative |
| R42 | *Solibacillus isronensis* | Planococcaceae | Bacillales | TSA | Positive |
| S15 | *Solibacillus silvestris* | Planococcaceae | Bacillales | LBA | Positive |
| R93 | *Streptomyces flavogriseus* | Streptomycetaceae | Streptomycetales | LBA | Positive |
| Ri17 | *Rhodanobacter spathiphylli* | Rhodanobacteraceae | Xanthomonadales | TSA | Negative |
| Ri21 | *Sphingobium mellinum* | Sphingomonadaceae | Sphingomonadales | TSA | Negative |
| Ri29 | *Pedobacter steynii* | Sphingobacteriaceae | Sphingobacteriales | TSA | Negative |
| Ri32 | *Pedobacter panaciterrae* | Sphingobacteriaceae | Sphingobacteriales | TSA | Negative |
| Ri55 | *Flavobacterium hauense* | Flavobacteriaceae | Flavobacteriales | LBA | Negative |
| Ri56 | *Nocardia coeliaca* | Nocardiaceae | Corynebacteriales | LBA | Positive |
| S13 | *Xanthomonas campestris* | Xanthomonadaceae | Xanthomonadales | R2A | Negative |
| S37 | *Pseudoxanthomonas suwonensis* | Xanthomonadaceae | Xanthomonadales | R2A | Negative |
| S19 | *Brevibacterium sediminis* | Brevibacteriaceae | Micrococcales | LBA | Positive |
| S55 | *Brevibacterium anseongense* | Brevibacteriaceae | Micrococcales | TSA | Positive |
| S25 | *Devosia riboflavina* | Devosiaceae | Rhizobiales | TSA | Negative |
| S29 | *Aeromonas hydrophila* | Aeromonadaceae | Enterobacterales | TSA | Negative |
| S52 | *Kaistia adipata* | Kaistiaceae | Rhizobiales | R2A | Negative |
| S64 | *Exiguobacterium artemiae* | Exiguobacteraceae | Exiguobacterales | TSA | Positive |
| S65 | *Cellulosimicrobium cellulans* | Promicromonosporaceae | Micrococcales | TSA | Positive |
| S71 | *Nocardioides zeae* | Nocardioidaceae | Propionibacteriales | TSA | Positive |
| Si1 | *Herbaspirillum rhizosphaerae* | Oxalobacteraceae | Burkholderiales | TSA | Negative |
