# Supplementary material for: The soil-borne white root rot pathogen Rosellinia necatrix expresses antimicrobial proteins during host colonization
Source: PLoS Pathog. 2024 Jan 18;20(1):e1011866. doi: 10.1371/journal.ppat.1011866 (PMC10796067; doi:10.1371/journal.ppat.1011866)
Supplement: S5 Table — (DOCX) [file ppat.1011866.s005.docx]

**S5 Table. Primers used in this study.**

| **Primer name** | **Oligonucleotide sequence (5’→3’)** | **Usage** |
| --- | --- | --- |
| RnGAPDH-F | TCAGCGACCAGGAGCTAGTCA | qPCR |
| RnGAPDH-R | TTGAAAAAATTTGGATTGAGCTCTAC | qPCR |
| coRUB-Fw | GAACAGTTTCTCACTGTTGAC | qPCR |
| coRUB-Rv | CGTGAGAACCATAAGTCACC | qPCR |
| RnITS-F | CTGTTCGAGCGTCATTTCAA | qPCR |
| RnITS-R | CCTACCTGATCCGAGGTCAA | qPCR |
| FUN_3304_NdeI_fw | ATCATATGATGATTTCCAACATCCTCCCTGTT | Protein production |
| FUN_3304_BamHI_rv | CCGGATCCTTAGCTGCTGCAGCCACCAAGC | Protein production |
| FUN_4580_NdeI_fw | ATCATATGATGAAGGCAACGATTTTGGACATCG | Protein production |
| FUN_4580_BamHI_rv | CCGGATCCTTATTCGCTGAAGGTGATGGTAACTG | Protein production |
| FUN_5751_NdeI_fw | ATCATATGATGCAGATCTTCACGACAGTCTTGGCCGT | Protein production |
| FUN_5751_BamHI_rv | AGCCGGATCCTCAGCTAGTCCCGGAGCAG | Protein production |
| FUN_9266_NdeI_fw | ATCATATGATGCGTGTCTCAGCTGCTCTCTTCG | Protein production |
| FUN_9266_BamHI_rv | CCGGATCCCTAAAGGGGGTCCGTCCGTC | Protein production |
| FUN_9480_NdeI_fw | ATCATATGATGAAGGCAACTCTGATCTCCGTCGCCGT | Protein production |
| FUN_9480_BamHI_rv | CCGGATCCTTACAGAAGAGCGGCGGCAGCCA | Protein production |
