# Supplementary material for: The soil-borne white root rot pathogen Rosellinia necatrix expresses antimicrobial proteins during host colonization
Source: PLoS Pathog. 2024 Jan 18;20(1):e1011866. doi: 10.1371/journal.ppat.1011866 (PMC10796067; doi:10.1371/journal.ppat.1011866)
Supplement: S6 Table — (DOCX) [file ppat.1011866.s006.docx]

**S6 Table. Annotation of BLAST and HMMER hits to effector FUN_004580.**

| **Accession ID^a^** | **Annotation^b^** | **Organism^c^** | **Query coverage** | **E value** | **Identity (%)^d^** |
| --- | --- | --- | --- | --- | --- |
| **BLAST** |  |  |  |  |  |
| XM_047979610.1 | Hypothetical protein | *Xylaria bambusicola* | 97% | 0.0 | 71.68% |
| XM_049263194.1 | Hypothetical protein | *Hypoxylon fragiforme* | 96% | 1E-151 | 57.95% |
| XM_051520408.1 | Hypothetical protein | *Durotheca rogersii* | 100% | 1E-144 | 54.59% |
| XM_047931715.1 | Hypothetical protein | *Daldinia caldariorum* | 97% | 2E-141 | 54.34% |
| XM_049244863.1 | Hypothetical protein | *Daldinia decipiens* | 97% | 1E-138 | 53.94% |
| XM_033578433.1 | Hypothetical protein | *Daldinia childiae* | 97% | 4E-137 | 53.06% |
| XM_049303926.1 | Hypothetical protein | *Daldinia loculata* | 97% | 2E-135 | 53.57% |
| XM_047960472.1 | Hypothetical protein | *Annulohypoxylon maeteangense* | 96% | 9E-135 | 51.28% |
| XM_048007749.1 | Hypothetical protein | *Daldinia vernicosa* | 97% | 1E-134 | 53.55% |
| XM_047993846.1 | Hypothetical protein | *Annulohypoxylon truncatum* | 97% | 4E-133 | 52.91% |
| XM_051460929.1 | Hypothetical protein | *Hypoxylon trugodes* | 96% | 9E-132 | 51.79% |
| XM_049311849.1 | Hypothetical protein | *Neoarthrinium moseri* | 100% | 7E-130 | 52.33% |
| XM_007830382.1 | Hypothetical protein | *Pestalotiopsis fici* | 100% | 3E-122 | 49.40% |
| XM_040853841.1 | Hypothetical protein | *Pseudomassariella vexata* | 99% | 4E-120 | 50.61% |
| XM_046099206.1 | Hypothetical protein | *Truncatella angustata* | 83% | 2E-105 | 60.57% |
| XM_046156523.1 | Hypothetical protein | *Microdochium trichocladiopsis* | 95% | 3E-105 | 46.56% |
| XM_018284675.1 | Hypothetical protein | *Pochonia chlamydosporia* | 95% | 2E-102 | 47.31% |
| XM_024896116.1 | Hypothetical protein | *Trichoderma citrinoviride* | 69% | 4E-100 | 56.54% |
| XM_006963277.1 | Hypothetical protein | *Trichoderma reesei* | 69% | 1E-99 | 56.99% |
| XM_024900564.1 | Hypothetical protein | *Trichoderma asperellum* | 96% | 7E-99 | 43.24% |
| XM_038885939.1 | Hypothetical protein | *Colletotrichum karsti* | 67% | 4E-98 | 56.20% |
| XM_018296053.1 | Hypothetical protein | *Colletotrichum higginsianum* | 68% | 1E-97 | 55.40% |
| XM_016786253.1 | Hypothetical protein | *Scedosporium apiospermum* | 94% | 1E-97 | 45.83% |
| XM_036632953.1 | Hypothetical protein | *Colletotrichum siamense* | 61% | 1E-96 | 58.23% |
| XM_045404242.1 | Hypothetical protein | *Colletotrichum gloeosporioides* | 61% | 2E-96 | 58.23% |
| XM_037316359.1 | Hypothetical protein | *Colletotrichum aenigma* | 61% | 2E-96 | 58.23% |
| XM_053175963.1 | Hypothetical protein | *Colletotrichum chrysophilum* | 61% | 2E-96 | 58.23% |
| XM_032030074.1 | Hypothetical protein | *Colletotrichum fructicola* | 61% | 2E-96 | 58.23% |
| XM_049274743.1 | Hypothetical protein | *Colletotrichum spaethianum* | 61% | 4E-96 | 60.00% |
| XM_043142060.1 | Hypothetical protein | *Ustilaginoidea virens* | 68% | 6E-96 | 55.20% |
| XM_046250215.1 | Hypothetical protein | *Ilyonectria robusta* | 67% | 2E-94 | 54.21% |
| XM_018809009.1 | Hypothetical protein | *Trichoderma gamsii* | 69% | 6E-94 | 52.63% |
| XM_044846055.1 | Hypothetical protein | *Fusarium poae* | 95% | 4E-93 | 42.65% |
| XM_007818233.1 | Hypothetical protein | *Metarhizium robertsii* | 95% | 7E-93 | 42.93% |
| XM_024922373.1 | Hypothetical protein | *Trichoderma harzianum* | 69% | 2E-92 | 53.55% |
| XM_036723064.1 | Hypothetical protein | *Colletotrichum truncatum* | 61% | 5E-92 | 57.43% |
| XM_035468007.1 | Hypothetical protein | *Geosmithia morbida* | 67% | 1E-91 | 54.58% |
| XM_014688387.1 | Hypothetical protein | *Metarhizium brunneum* | 95% | 1E-91 | 42.68% |
| XM_009264636.1 | Hypothetical protein | *Fusarium pseudograminearum* | 68% | 2E-91 | 51.97% |
| XM_003651639.1 | Hypothetical protein | *Thermothielavioides terrestris* | 96% | 4E-91 | 43.73% |
| XM_047979610.1 | Hypothetical protein | *Xylaria bambusicola* | 97% | 0.0 | 71.68% |
| XM_049263194.1 | Hypothetical protein | *Hypoxylon fragiforme* | 96% | 1E-151 | 57.95% |
| XM_051520408.1 | Hypothetical protein | *Durotheca rogersii* | 100% | 1E-144 | 54.59% |
| XM_047931715.1 | Hypothetical protein | *Daldinia caldariorum* | 97% | 2E-141 | 54.34% |
| XM_003651639.1 | Hypothetical protein | *Thermothielavioides terrestris* | 96% | 4E-91 | 43.73% |
| XM_025729660.1 | Hypothetical protein | *Fusarium venenatum* | 68% | 1E-90 | 51.25% |
| XM_008095090.1 | Hypothetical protein | *Colletotrichum graminicola* | 61% | 3E-90 | 56.22% |
| XM_014092778.1 | Hypothetical protein | *Trichoderma atroviride* | 69% | 3E-90 | 52.28% |
| XM_049285132.1 | Hypothetical protein | *Colletotrichum lupini* | 60% | 4E-90 | 56.68% |
| CP077948.1 | Hypothetical protein | *Colletotrichum gigasporum* | 92% | 1E-89 | 45.48% |
| XM_053195163.1 | Hypothetical protein | *Colletotrichum fioriniae* | 60% | 6E-89 | 56.56% |
| OW971920.1 | Hypothetical protein | *Trichoderma pseudokoningii* | 67% | 1E-88 | 56.57% |
| XM_031165144.1 | Hypothetical protein | *Fusarium coffeatum* | 68% | 2E-88 | 51.25% |
| XM_006691392.1 | Hypothetical protein | *Thermochaetoides thermophila* | 94% | 7E-88 | 41.45% |
| CP021290.1 | Hypothetical protein | *Trichoderma reesei* | 66% | 1E-87 | 57.88% |
| CP040201.1 | Hypothetical protein | *Trichoderma reesei* | 66% | 1E-87 | 57.88% |
| CP020724.1 | Hypothetical protein | *Trichoderma reesei* | 66% | 1E-87 | 57.88% |
| CP020875.1 | Hypothetical protein | *Trichoderma reesei* | 66% | 1E-87 | 57.88% |
| CP021304.1 | Hypothetical protein | *Trichoderma reesei* | 66% | 1E-87 | 57.88% |
| CP021297.1 | Hypothetical protein | *Trichoderma reesei* | 66% | 1E-87 | 57.88% |
| CP016232.1 | Hypothetical protein | *Trichoderma reesei* | 66% | 1E-87 | 57.88% |
| CP021311.1 | Hypothetical protein | *Trichoderma reesei* | 66% | 1E-87 | 57.88% |
| CP084946.1 | Hypothetical protein | *Trichoderma asperellum* | 94% | 1E-87 | 43.11% |
| XM_022625650.1 | Hypothetical protein | *Colletotrichum orchidophilum* | 68% | 3E-87 | 51.97% |
| XM_011318840.1 | Hypothetical protein | *Fusarium graminearum* | 68% | 4E-87 | 51.97% |
| XM_046123457.1 | Hypothetical protein | *Fusarium flagelliforme* | 68% | 4E-87 | 51.60% |
| CP072834.1 | Hypothetical protein | *Trichoderma asperellum* | 94% | 7E-87 | 42.75% |
| XM_014095116.1 | Hypothetical protein | *Trichoderma virens* | 69% | 1E-86 | 52.13% |
| XM_053146149.1 | Hypothetical protein | *Fusarium falciforme* | 68% | 1E-86 | 50.18% |
| XM_028643441.1 | Hypothetical protein | *Verticillium nonalfalfae* | 61% | 2E-86 | 54.66% |
| XM_003350974.1 | Hypothetical protein | *Sordaria macrospora* | 68% | 3E-86 | 49.64% |
| XM_053050566.1 | Hypothetical protein | *Fusarium keratoplasticum* | 67% | 1E-85 | 50.55% |
| XM_046270538.1 | Hypothetical protein | *Fusarium solani* | 67% | 1E-85 | 50.55% |
| XM_035474047.1 | Hypothetical protein | *Colletotrichum scovillei* | 60% | 1E-85 | 56.68% |
| CP083246.1 | Hypothetical protein | *Epichloe scottii* | 66% | 1E-84 | 53.73% |
| CP101605.1 | Hypothetical protein | *Ustilaginoidea virens* | 65% | 2E-84 | 55.43% |
| CP072756.1 | Hypothetical protein | *Ustilaginoidea virens* | 65% | 2E-84 | 55.43% |
| CP049928.1 | Hypothetical protein | *Ustilaginoidea virens* | 65% | 2E-84 | 55.43% |
| XM_018276255.1 | Hypothetical protein | *Pseudogymnoascus verrucosus* | 93% | 4E-84 | 40.57% |
| CP079833.1 | Hypothetical protein | *Fusarium graminearum* | 68% | 9E-84 | 51.97% |
| XM_003003371.1 | Hypothetical protein | *Verticillium alfalfae* | 61% | 9E-84 | 54.66% |
| CP064806.1 | Hypothetical protein | *Epichloe typhina subsp. clarkii* | 67% | 1E-83 | 51.25% |
| CP003010.1 | Hypothetical protein | *Thermothielavioides terrestris* | 95% | 1E-83 | 43.65% |
| XM_008086879.1 | Hypothetical protein | *Glarea lozoyensis* | 68% | 1E-83 | 49.47% |
| CP117785.1 | Hypothetical protein | *Colletotrichum graminicola* | 65% | 2E-83 | 54.72% |
| CP019475.1 | Hypothetical protein | *Colletotrichum lupini* | 60% | 4E-83 | 56.68% |
| XM_009858316.1 | Hypothetical protein | *Neurospora tetrasperma* | 68% | 4E-83 | 49.28% |
| XM_046198521.1 | Hypothetical protein | *Fusarium redolens* | 69% | 6E-83 | 51.42% |
| CP100344.1 | Hypothetical protein | *Epichloe festucae* | 66% | 1E-82 | 53.36% |
| CP031387.1 | Hypothetical protein | *Epichloe festucae* | 66% | 1E-82 | 53.36% |
| CP098298.1 | Hypothetical protein | *Epichloe typhina subsp. poae* | 67% | 2E-82 | 51.61% |
| CP084938.1 | Hypothetical protein | *Trichoderma atroviride* | 67% | 2E-82 | 52.71% |
| XM_047982779.1 | Hypothetical protein | *Purpureocillium takamizusanense* | 69% | 2E-82 | 48.41% |
| XM_001907097.1 | Hypothetical protein | *Podospora anserina* | 97% | 3E-82 | 37.78% |
| XM_040954205.1 | Hypothetical protein | *Penicillium solitum* | 68% | 3E-82 | 49.82% |
| CP069151.1 | Hypothetical protein | *Verticillium nonalfalfae* | 65% | 4E-82 | 53.61% |
| **HMMER** |  |  |  |  |  |
| A0A5N6KR04_9ROSI | Hypothetical protein | *Carpinus fangiana* | 59,2% | 1.8E-66 | 41,1% |

**^a^**Database accession ID where subject was deposited.

**^b^**Annotation of the best hit using BLAST (tblastn).

**^c^**Organism where the homolog was annotated.

**^d^**Percentage of identity of the query.
