# Supplementary material for: The soil-borne white root rot pathogen Rosellinia necatrix expresses antimicrobial proteins during host colonization
Source: PLoS Pathog. 2024 Jan 18;20(1):e1011866. doi: 10.1371/journal.ppat.1011866 (PMC10796067; doi:10.1371/journal.ppat.1011866)
Supplement: S7 Table — (DOCX) [file ppat.1011866.s007.docx]

**S7 Table. Annotation of BLAST and HMMER hits to effector FUN_011519.**

| **Accession ID^a^** | **Annotation^b^** | **Organism^c^** | **Query coverage** | **E value** | **Identity (%)^d^** |
| --- | --- | --- | --- | --- | --- |
| **BLAST and HMMER** | | | | | |
| XM_007838424.1 | Hypothetical protein | *Pestalotiopsis fici* | 100% | 6E-10 | 68.57% |
| XM_014319017.1 | Hypothetical protein | *Grosmannia clavigera* | 100% | 1E-09 | 71.43% |
| XM_006668457.1 | Hypothetical protein | *Cordyceps militaris* | 100% | 1E-09 | 71.43% |
| XM_032032777.1 | Hypothetical protein | *Colletotrichum fructicola* | 100% | 2E-09 | 65.71% |
| XM_024655518.1 | Hypothetical protein | *Sordaria macrospora* | 100% | 1E-08 | 68.57% |
| XM_003659946.1 | Hypothetical protein | *Thermothelomyces thermophilus* | 100% | 2E-08 | 71.43% |
| XM_048011148.1 | Hypothetical protein | *Daldinia vernicosa* | 100% | 2E-08 | 62.86% |
| XM_046108154.1 | Hypothetical protein | *Truncatella angustata* | 100% | 6E-08 | 60.00% |
| XM_051511661.1 | Hypothetical protein | *Durotheca rogersii* | 100% | 6E-08 | 62.86% |
| XM_001907105.1 | Hypothetical protein | *Podospora anserina* | 100% | 7E-08 | 65.71% |
| XM_046158086.1 | Hypothetical protein | *Microdochium trichocladiopsis* | 100% | 9E-08 | 60.00% |
| XM_047937750.1 | Hypothetical protein | *Daldinia caldariorum* | 100% | 1E-07 | 60.00% |
| XM_028614903.1 | Hypothetical protein | *Sodiomyces alkalinus* | 100% | 2E-07 | 68.57% |
| XM_051457076.1 | Hypothetical protein | *Hypoxylon trugodes* | 100% | 4E-07 | 65.71% |
| XM_049244688.1 | Hypothetical protein | *Daldinia decipiens* | 100% | 4E-07 | 65.71% |
| XM_014686345.1 | Hypothetical protein | *Metarhizium brunneum* | 100% | 5E-07 | 60.00% |
| XM_003007773.1 | Hypothetical protein | *Verticillium alfalfae* | 100% | 2E-06 | 60.00% |
| XM_003651533.1 | Hypothetical protein | *Thermothielavioides terrestris* | 97% | 3E-11 | 79.41% |
| XM_046265423.1 | Hypothetical protein | *Emericellopsis atlantica* | 97% | 2E-09 | 73.53% |
| XM_008095371.1 | Hypothetical protein | *Colletotrichum graminicola* | 97% | 5E-09 | 70.27% |
| XM_040863558.1 | Hypothetical protein | *Pseudomassariella vexata* | 97% | 7E-09 | 67.65% |
| XM_960199.2 | Hypothetical protein | *Neurospora crassa* | 97% | 2E-08 | 76.47% |
| XM_047972197.1 | Hypothetical protein | *Xylaria bambusicola* | 97% | 4E-08 | 64.71% |
| XM_049302929.1 | Hypothetical protein | *Daldinia loculata* | 97% | 2E-07 | 64.71% |
| XM_049293600.1 | Hypothetical protein | *Colletotrichum lupini* | 97% | 2E-07 | 67.57% |
| XM_035467298.1 | Hypothetical protein | *Geosmithia morbida* | 97% | 4E-07 | 61.76% |
| XM_022620490.1 | Hypothetical protein | *Colletotrichum orchidophilum* | 97% | 4E-07 | 62.16% |
| XM_044863015.1 | Hypothetical protein | *Hirsutella rhossiliensis* | 97% | 7E-07 | 67.65% |
| XM_047991640.1 | Hypothetical protein | *Purpureocillium takamizusanense* | 97% | 1E-06 | 70.59% |
| XM_007808999.1 | Hypothetical protein | *Metarhizium acridum* | 97% | 2E-06 | 61.76% |
| XM_009653120.1 | Hypothetical protein | *Verticillium dahliae* | 97% | 2E-06 | 61.76% |
| XM_046253466.1 | Hypothetical protein | *Ilyonectria robusta* | 97% | 3E-06 | 61.76% |
| XM_053054048.1 | Hypothetical protein | *Fusarium keratoplasticum* | 97% | 5E-05 | 55.88% |
| XM_031161470.1 | Hypothetical protein | *Fusarium coffeatum* | 97% | 6E-05 | 55.88% |
| XM_025733484.1 | Hypothetical protein | *Fusarium venenatum* | 97% | 1E-04 | 55.88% |
| XM_023579291.1 | Hypothetical protein | *Fusarium fujikuroi* | 97% | 2E-04 | 52.94% |
| XM_046198815.1 | Hypothetical protein | *Fusarium redolens* | 97% | 2E-04 | 52.94% |
| XM_053149576.1 | Hypothetical protein | *Fusarium falciforme* | 97% | 3E-04 | 55.88% |
| XM_046283930.1 | Hypothetical protein | *Fusarium solani* | 97% | 4E-04 | 55.88% |
| XM_029896370.1 | Hypothetical protein | *Pyricularia pennisetigena* | 94% | 2E-08 | 72.73% |
| XM_003720841.1 | Hypothetical protein | *Pyricularia oryzae* | 94% | 9E-08 | 69.70% |
| XM_014087113.1 | Hypothetical protein | *Trichoderma atroviride* | 94% | 4E-07 | 63.64% |
| XM_024898021.1 | Hypothetical protein | *Trichoderma citrinoviride* | 94% | 5E-07 | 66.67% |
| XM_031130497.1 | Hypothetical protein | *Pyricularia grisea* | 94% | 1E-06 | 69.70% |
| XM_024548995.1 | Hypothetical protein | *Trichoderma gamsii* | 94% | 3E-06 | 60.61% |
| XM_024901739.1 | Hypothetical protein | *Trichoderma asperellum* | 94% | 8E-06 | 60.61% |
| XM_024919849.1 | Hypothetical protein | *Trichoderma harzianum* | 94% | 1E-05 | 66.67% |
| XM_043141820.1 | Hypothetical protein | *Ustilaginoidea virens* | 91% | 6E-07 | 65.62% |
| XM_009229853.1 | Hypothetical protein | *Gaeumannomyces tritici* | 91% | 5E-06 | 59.38% |
| CP023323.1 | Hypothetical protein | *Cordyceps militaris* | 88% | 2E-07 | 74.19% |
| CP003010.1 | Hypothetical protein | *Thermothielavioides terrestris* | 88% | 3E-07 | 77.42% |
| XM_040822091.1 | Hypothetical protein | *Metarhizium album* | 88% | 2E-06 | 70.97% |
| XM_049264651.1 | Hypothetical protein | *Hypoxylon fragiforme* | 88% | 3E-06 | 58.06% |
| XM_009857960.1 | Hypothetical protein | *Neurospora tetrasperma* | 88% | 4E-06 | 74.19% |
| CP045886.1 | Hypothetical protein | *Beauveria bassiana* | 88% | 4E-06 | 61.29% |
| CP098299.1 | Hypothetical protein | *Epichloe typhina* | 88% | 4E-06 | 67.74% |
| CP098307.1 | Hypothetical protein | *Epichloe typhina* | 88% | 4E-06 | 67.74% |
| CP064797.1 | Hypothetical protein | *Epichloe typhina* | 88% | 5E-06 | 67.74% |
| CP003002.1 | Hypothetical protein | *Thermothelomyces thermophilus* | 88% | 8E-06 | 70.97% |
| CP031390.1 | Hypothetical protein | *Epichloe festucae* | 88% | 2E-05 | 67.74% |
| CP100347.1 | Hypothetical protein | *Epichloe festucae* | 88% | 2E-05 | 67.74% |
| XM_031143259.1 | Hypothetical protein | *Thyridium curvatum* | 88% | 2E-05 | 64.52% |
| CP098267.1 | Hypothetical protein | *Epichloe bromicola* | 88% | 2E-05 | 67.74% |
| CP064805.1 | Hypothetical protein | *Epichloe typhina* | 88% | 2E-05 | 67.74% |
| CP049927.1 | Hypothetical protein | *Ustilaginoidea virens* | 88% | 2E-05 | 64.52% |
| CP072755.1 | Hypothetical protein | *Ustilaginoidea virens* | 88% | 2E-05 | 64.52% |
| CP101604.1 | Hypothetical protein | *Ustilaginoidea virens* | 88% | 2E-05 | 64.52% |
| CP099637.1 | Hypothetical protein | *Epichloe amarillans* | 88% | 2E-05 | 67.74% |
| CP098274.1 | Hypothetical protein | *Epichloe elymi* | 88% | 2E-05 | 67.74% |
| CP077954.1 | Hypothetical protein | *Colletotrichum gigasporum* | 88% | 3E-05 | 64.52% |
| XM_007825388.2 | Hypothetical protein | *Metarhizium robertsii* | 88% | 3E-05 | 61.29% |
| XM_035474232.1 | Hypothetical protein | *Colletotrichum scovillei* | 88% | 4E-05 | 64.71% |
| CP086364.1 | Hypothetical protein | *Purpureocillium takamizusanense* | 88% | 5E-05 | 70.97% |
| XM_040763701.1 | Hypothetical protein | *Sporothrix brasiliensis* | 88% | 6E-05 | 67.74% |
| AB669186.1 | Hypothetical protein | *Colletotrichum orbiculare* | 88% | 7E-05 | 61.29% |
| CP096782.1 | Hypothetical protein | *Nigrospora oryzae* | 88% | 7E-05 | 64.52% |
| CP069148.1 | Hypothetical protein | *Verticillium nonalfalfae* | 88% | 9E-05 | 58.06% |
| CP069139.1 | Hypothetical protein | *Verticillium nonalfalfae* | 88% | 9E-05 | 58.06% |
| CP019480.1 | Hypothetical protein | *Colletotrichum lupini* | 88% | 1E-04 | 64.71% |
| XM_018304892.1 | Hypothetical protein | *Colletotrichum higginsianum* | 88% | 2E-04 | 58.82% |
| CP010982.1 | Hypothetical protein | *Verticillium dahliae* | 88% | 3E-04 | 58.06% |
| CP009079.1 | Hypothetical protein | *Verticillium dahliae* | 88% | 3E-04 | 58.06% |
| CP058936.1 | Hypothetical protein | *Metarhizium brunneum* | 88% | 3E-04 | 61.29% |
| LR026964.1 | Hypothetical protein | *Podospora comata* | 88% | 4E-04 | 64.52% |
| CP071116.1 | Hypothetical protein | *Trichoderma virens* | 85% | 7E-06 | 66.67% |
| XM_014102688.1 | Hypothetical protein | *Trichoderma virens* | 85% | 2E-05 | 66.67% |
| CP084938.1 | Hypothetical protein | *Trichoderma atroviride* | 85% | 4E-05 | 63.33% |
| CP091464.1 | Hypothetical protein | *Pyricularia oryzae* | 85% | 7E-05 | 66.67% |
| CP084944.1 | Hypothetical protein | *Trichoderma asperellum* | 85% | 8E-05 | 60.00% |
| CP072831.1 | Hypothetical protein | *Trichoderma asperellum* | 85% | 1E-04 | 60.00% |
| CP034210.1 | Hypothetical protein | *Pyricularia oryzae* | 85% | 2E-04 | 66.67% |
| CP060336.1 | Hypothetical protein | *Pyricularia oryzae* | 85% | 2E-04 | 66.67% |
| CP099702.1 | Hypothetical protein | *Pyricularia oryzae* | 85% | 2E-04 | 66.67% |
| CP050926.1 | Hypothetical protein | *Pyricularia oryzae* | 85% | 2E-04 | 66.67% |
| CP075865.1 | Hypothetical protein | *Trichoderma simmonsii* | 85% | 2E-04 | 66.67% |
| CP071108.1 | Hypothetical protein | *Trichoderma virens* | 85% | 2E-04 | 66.67% |
| OW971923.1 | Hypothetical protein | *Trichoderma pseudokoningii* | 85% | 3E-04 | 66.67% |
| CP021293.1 | Hypothetical protein | *Trichoderma reesei* | 85% | 3E-04 | 66.67% |
| CP016235.1 | Hypothetical protein | *Trichoderma reesei* | 85% | 3E-04 | 66.67% |
| CP021307.1 | Hypothetical protein | *Trichoderma reesei* | 85% | 3E-04 | 66.67% |

**^a^**Database accession ID where subject was deposited.

**^b^**Annotation of the best hit using BLAST (tblastn).

**^c^**Organism where the homolog was annotated.

**^d^**Percentage of identity of the query.
