# Supplementary material for: The soil-borne white root rot pathogen Rosellinia necatrix expresses antimicrobial proteins during host colonization
Source: PLoS Pathog. 2024 Jan 18;20(1):e1011866. doi: 10.1371/journal.ppat.1011866 (PMC10796067; doi:10.1371/journal.ppat.1011866)
Supplement: Attachment — Submitted filename: response to reviewers.pdf [file ppat.1011866.s008.pdf]

Dear editor,

Thank you very much for considering our manuscript 'The soil-borne white root rot pathogen *Rosellinia necatrix* expresses antimicrobial proteins during host colonization' for publication in PLoS Pathogens. In this letter, we detail our responses to the reviewers' comments. All changes are highlighted (in yellow) in our revised submission. We hope that our changes satisfy the reviewers and yourself, and we are looking forward to your response.

With kind regards,

Bart Thomma, on behalf of all authors

Editor comments

Editor: In addition to the suggested experiments by reviewer 1 and if experimentally possible provide a phenotypic assessment of loss-of-function mutants in *R. necatrix* for FUN_004580 and FUN_011519 or a technical or scientific justification on why these mutants had not been generated and tested.

Author response: Although two studies published about 20 years ago have reported transformation of *R. necatrix* (Kanematsu et al. 2004, Phytopathology 94: 561–568; Aimi et al., 2005, Mycoscience 46: 27–31), these protocols have gained very little traction and have certainly not become routine procedures. In fact, no successful recent transformation attempts have been reported. We have tried several times to generate knockouts in *R. necatrix*, but despite our extensive experience with fungal transformation involving a range of fungal species (*Verticillium* spp., *Cladosporium fulvum*, *Zymoseptoria tritici*, *Magnaporthe oryzae*, *Fusarium* spp.), without success. Therefore, no transformation/targeted deletion system is available that allows the phenotypic assessment of loss-of-function in *R. necatrix* isolate R18 for FUN_004580 and FUN_011519.

Editor: In the revised manuscript, please make sure to emphasise that predicted secreted proteins are in the focus of this study or provide experimental evidence for their secretion.

Author response: We have now emphasized at several instances in the manuscript that proteins are predicted to be secreted, and we also report the (very high) prediction confidence scores now.

==

Reviewer 1

Major Issues: Key Experiments Required for Acceptance

Reviewer #1: 1.) Figure 5: In addition to the heat inactivation, it would be desirable to see whether the antimicrobial activity of the culture filtrate is also protease-sensitive. This could be tested with a selected number of bacteria that are inhibited in growth by the culture filtrate (e.g. B. drentensis, A. denitrificans and S. mellinum).

Author response: We avoid the use of proteases as they are usually in buffers with SDS and/or EDTA that affect bacterial growth and once we add this to the extract we cannot remove these chemicals. However, to address the question whether the antimicrobial activity is likely of proteinaceous nature, or more likely due to the activity of metabolites or other types of small molecules, we used spin columns with 3 kDa cut-off and showed that cultures filtrates that passed through the spin column are no longer inhibiting the growth of the bacteria *Bacillus drentensis* and *Flavobacterium huaense*. This suggests that the antimicrobial activity is mediated by proteins.

Reviewer #1: 2.) Figure 9: As for Figure 5, I would request to test for the heat- and protease-sensitivity of the antimicrobial effect of the two selected R. necatrix effectors using one or two representative fungal/bacterial cases as additional controls.

Author response: We have now included data for heat-inactivated proteins and spin-columns filtrates as explained above to demonstrate that the antimicrobial activity is likely of proteinaceous origin.

Reviewer #1: 3.) Figure 11: Similar to my comments to Figures 5 and 9, I wonder what the effect of pre-treatment of the cotton seeds with heat-killed bacteria would be. Also, as an additional control, preincubation with bacteria that likely would not inhibit R. necatrix would be desired. In other words: Is the bacterial inhibition shown in this Figure an effect specific for the bacteria chosen or would any bacterium inhibit growth of the fungal phytopathogen?

Author response: We have not performed experiments with heat-killed bacteria because this will lead to cellular collapse and the release of cellular constituents into the mixture. However, in Figure 11 we now show that four non-antagonistic bacterial species are not able to protect cotton seedlings from *Rosellinia* disease, suggesting that it is a specific activity mediated by particular bacterial strains, rather than the activation of MAMP-triggered immunity, that leads to the protective effect.

Minor Issues:

Reviewer #1: 1.) Table 1: Could the authors provide BUSCO values for all nine sequenced R. necatrix strains?

Author response: We have now included BUSCO scores for all assemblies in Table 1.

Reviewer #1: 2.) Effector prediction: As the authors have available genomic sequence data from nine R. necatrix strains, it would be desirable to see a comparison across these strains regarding conservation of the predicted effectors (both presence/absence and SNPs). Of course there is the caveat of incomplete genome assemblies for eight of the strains, but the quality parameters look rather decent.

Author response: We compared effector catalogs across the assemblies and show the data on presence-absence variation as well as on sequence variation in Supplemental Table 2.

Reviewer #1: 3.) Figure 3: I do not know the Realphy tool, but would it be possible to derive bootstrap values or any other measure of reliability of the phylogenetic tree?

Author response: Indeed, bootstrap analysis was conducted with 1,000 replicates to assess the robustness of the phylogenetic tree. Bootstrap values were higher than 90% indicating that branches are well supported. This information has now been added to the figure legend.

Reviewer #1: 4.) Figure 5: It would be good to know on how many replicates/experiments the data shown in Figure 5 are based and what the error bars show. Moreover, I cannot see a blue line for some of the bacteria (e.g. A. denitrificans, but also others). If in these cases the 4-day time point was omitted, this should be indicated in the Figure legend.

Author response: The experiment was performed twice with three replicates (n=3) and the error bar shows the standard deviation based on the replicates (n=3), we added this

information to the figure legend. For all the bacteria all time points were tested but in some cases lines overlap.

Reviewer #1: 5.) Figure 10: I assume the right column of photographs is a negative control of bacterial growth in the absence of R. necatrix? Should be mentioned in the Figure legend. What does n=5 mean here? We only see one set of photographs. If five replicates exist, could the authors provide quantitative data for this experiment by quantifying the area of fungal growth and calculate inhibition?

Author response: We now added the description for the right column in the figure legend as suggested. Indeed, we performed 5 replicates and only took one representative picture, given that the variation was absolutely minimal among the replicates and show very obvious phenotypic differences. We believe that quantification adds little value as little can be inferred from such values.

Reviewer #1: 6.) Figure 11: What does "Mix" mean here? All four bacteria mixed? Should be mentioned in the Figure legend.

Author response: We added the description for "mix" in the figure legend as suggested.

Reviewer #1: 7.) Materials and Methods: The origin of the fungal isolates should be more clearly stated. When and where ("Mexico" is a rather broad description) were these collected?

Author response: We have now included sampling information for the isolates.

Reviewer #1: 8.) Line 263: "suggest" should read "suggests"

Author response: We have made the change accordingly.

Reviewer #1: 9.) Line 282: Not only fungi, but also oomycetes (e.g. Phytophthora!). Maybe better "filamentous phytopathogens (fungi and oomycetes)

Author response: We changed the phrase accordingly.

Reviewer #1: 10.) Line 316: "cluster" should read "clusters"

Author response: We have made the change accordingly.

Reviewer #1: 11.) Line 374 ff. - Prediction of antimicrobial effector candidates: What is known about the antimicrobial mechanism of AFP1 and AcAMP2? How do they act? Maybe add to Discussion.

Author response: We have included a discussion section on the modes of action of AFP1 and AcAMP1.

Reviewer #1: 12.) Line 473: "quantify" should read "quantified"

Author response: We have made the change accordingly.

Reviewer #1: 13.) Line 552: Delete “a” in front of “specific”

Author response: We have made the change accordingly.

Reviewer #1: 14.) Line 736: Please provide the R. necatrix gene identifier for the GSAPDH reference gene

Author response: We have included the gene ID as suggested.

Reviewer #1: 15.) Line 997: “Plant Phytologist” should read “New Phytologist”

Author response: We have made the change accordingly.

==

Reviewer 2

Major Issues: Key Experiments Required for Acceptance

Reviewer #2: 1. The authors make the claim that the genome does not appear to experience a two-speed genome. While this may be true, it appears to be confounded with a very low rate of repetitive sequences in the genome. Perhaps this should be pointed out more strongly, as this result is very different than many other plant pathogen genomes. Has any other group identified a two-speed genome with a plant pathogen with this low of a repeat content?

Author response: A similar observation was made for the genome of Ramularia leaf spot fungus *Ramularia collo-cygni* (Stam et al., 2018, Genome Biology and Evolution 10:3243–3249), which is now added to the discussion.

Reviewer #2: 2. The authors state: “This suggest that geographic origin of the strains correlates with their phylogenetic relationship.” This statement seems to be confounded with host sampling, therefore it seems to have limited scope.

Author response: We have eliminated the statement.

Reviewer #2: 3. “This analysis revealed that almost 40 percent of the effector candidates are structurally unique, while the remaining effectors could be assigned to a total of 31 clusters.” How many of the effector candidates had reasonable folds predicted by AlphaFold2? What was the distribution of proteins in the MSA for these effectors? The unique status may be due to insufficient training data sets for the MSA component of the analysis. Was any clustering performed on the proteins prior to analysis, perhaps with OrthoMCL?

Author response: We have included further analysis to investigate whether subsets of *R. necatrix* effectors display similar fold conservation, showing that the fold prediction of the *R. necatrix* effector catalogue is generally robust. We also performed similarity clustering of the predicted effector folds, showing that almost 40% of the effector candidates are structurally unique.

Reviewer #2: 4. Why were candidate antimicrobial peptides not also tested against Rosellinia necatrix as a control?

Author response: We have now included *R. necatrix* in Figure 8 and show that the proteins do not affect *R. necatrix in vitro*.

Reviewer #2: 5. Figure 11. To what extent is this observation simply PTI induction versus direct antagonistic activity? The experiment lacks negative controls.

Author response: We have now included non-antagonistic bacteria and show that only the antagonistic ones reduce Rosellinia disease. This finding strongly suggests disease reduction is based on antagonism rather than on PTI induction.

Minor Issues:

Reviewer #2: 1. Did you investigate why some BUSCO genes were missing? Were these a subset of proteins that are more variable? Did the annotation pipeline miss genes that are present in the genome?

Author response: We used as reference dataset the genes from the Ascomycota (taxonomical order) and therefore is possible that some genes are not present or are different in *Rosellinia necatrix* that belongs to the Xylariales family. This family contains a limited amount of genetic information and therefore differences in the dataset at the order level could easily occur. Using the Ascomycota reference set is a common strategy to assess completeness of genomes of species that belong to this order, and it is well known that gapless genome assemblies for various fungi, (e.g. *Verticillium dahliae*) never reach 100% for this reason (Faino et al., 2015 mBio 6(4):e00936-15).

Reviewer #2: 2. Lines 577-579 "In this study, we show that the soil-borne pathogen Rosellinia necatrix similarly secretes effector proteins during host colonization that possess selective antimicrobial activity." No experiment was performed to show that these effector proteins were secreted, therefore this claim cannot be made. Throughout, it should be clear that they are predicted secreted proteins unless further evidence is provided.

Author response: We have changed the phrase to stress that they are predicted secreted proteins. Additionally, in Table 3 we included a column with predicted signal peptide probability underpin that they are very likely to be secreted.

==

Reviewer 3

Minor Issues:

Reviewer #3: Line 161 explain the abbreviation HMW at first mention

Author response: We have explained the abbreviation accordingly.

Reviewer #3: Lines 263-264. I partially disagree with this suggestion since phylogenetic relationship could also correlate with the host plant.

Author response: We have deleted the phrase.

Reviewer #3: Line 283 Figure 3A. Please consider using better to distinguish colors for Deletion and Translocation

Author response: We have made changes accordingly.

Reviewer #3: Lines 381 and 383: assess instead of asses

Author response: We have made the change accordingly.

Reviewer #3: Line 491 and 524 bacterial species

Author response: We have made the change accordingly.

Reviewer #3: Line 493 Fig. 10 instead of Fig. 9

Author response: We have made the change accordingly.

Reviewer #3: Line 497 Fig. 10 instead of Fig. 8

Author response: We have made the change accordingly.

Reviewer #3: Line 509 “the fungus can promote the suppression of disease development” sounds clumsy to me. How about “the fungus can inhibit disease development”?

Author response: We have rephrased the sentence as suggested.

Reviewer #3: Lines 513 and 520: Fig. 11 instead of Fig. 10

Author response: We have made the change accordingly.

Reviewer #3: Line 552 “towards specific”, delete “a”

Author response: We have made the change accordingly.

Reviewer #3: Lines 561-564 The word complement seems to be used with an unusual meaning for biologists. Normally, if two proteins can complement each other, the one can fulfill the function of the other in the others absence. Here, the activities of the two proteins are clearly different. I suggest rephrasing.

Author response: We have rephrased accordingly.

Reviewer #3: Lines 616-619 In this sentence, I can understand each half sentence, however, it is not clear to me, why the second half sentence would result from the first. It would be helpful if the authors could clarify this in the text.

Author response: We have clarified in the text as follows: “The five largest clusters identified were mainly driven by sequence conservation rather than structural similarity, suggesting that effector family expansion based on structural similarity played only a minor role in *R. necatrix* effector evolution”.
