# Supplementary material for: The soil-borne white root rot pathogen Rosellinia necatrix expresses antimicrobial proteins during host colonization
Source: PLoS Pathog. 2024 Jan 18;20(1):e1011866. doi: 10.1371/journal.ppat.1011866 (PMC10796067; doi:10.1371/journal.ppat.1011866)
Supplement: Attachment — Submitted filename: Replies to referee queries.pdf [file ppat.1011866.s009.pdf]

Reviewer #1: 1.) I appreciate that the robustness of the tree shown in Figure 3 was assessed by 1000 bootstrap replicates. Typically, these only in part support the original tree; that is why usually "bootstrap values" (e.g. % support) are given at branches of phylogenetic trees. These should be included in the tree.

Response: the bootstrap values were 100 for all branches (100% supported), so now we highlight in red the branches, and in the figure legend we included the colour explanation as follows: branches with maximum (100%) bootstrap support are indicated in red.

Reviewer #1: 2.) The legend of Figure 5B is somewhat unclear. Do the black, light green and pink lines relate to sampling at 4, 7 and 9 days of fungal growth? If so, the legend needs adjustment. A color-coded legend in the Figure would also help readers and is suggested to be included (also true for Figure 9).

Response: The black, light green and pink lines relate to cultures filtrates that were sample at 4, 7 and 9 days of fungal growth and passed through spin columns with 3 kDa cut-off. To clarify this, we adjusted the figure legend as follows: Growth of *Bacillus drentensis* and *Flavobacterium hauense* was measured in culture filtrates collected after 4 (red), 7 (grey) and 9 (green) days of fungal growth, and additionally, in culture filtrates passed through spin-columns with 3 kDa cut-off after 4 (black), 7 (light green) and 9 (pink) days of fungal growth. We also include a color-coded legend within Figures 5 and 9 as requested.
